# A “fishy” situation, rare pathogen and presentation in prosthetic valve infective endocarditis

**DOI:** 10.1016/j.heliyon.2024.e32383

**Published:** 2024-06-04

**Authors:** Nicole Schtupak, Patrick Kenney, Darko Pucar, Linda Godinez, Jodi-Ann Chin, Kristen Selema, Dipan Uppal, Antonio Lewis, Marcelo Helguera

**Affiliations:** a Cleveland Clinic Florida, Department of Hospital Medicine, 2950 Cleveland Clinic Blvd., Weston, FL, 33331, United States; b Cleveland Clinic Florida, Department of Infectious Diseases, 2950 Cleveland Clinic Blvd., Weston, FL, 33331, United States; c Cleveland Clinic Florida, Department of Cardiology, 2950 Cleveland Clinic Blvd., Weston, FL, 33331, United States; d Cleveland Clinic Florida, Department of Radiology, 2950 Cleveland Clinic Blvd., Weston, FL, 33331, United States

**Keywords:** Lactococcus garviae, Endocarditis, Bacteremia, Symptomatic anemia, Gastrointestinal disease, Gastrointestinal disorders, Radiology, Cardiology, Prosthetic valve

## Abstract

*Lactococcus garviae* (*L. garviae*) is a gram-positive coccus belonging to the Streptococcaceae family. While primarily a pathogen in fish farms causing hemorrhagic sepsis, it can act as a rare opportunistic pathogen in humans. A 2021 case report by Bravo et al. documented less than 30 cases of infective endocarditis caused by *L. garviae* worldwide at that time [1]. This case report describes the 27th documented case globally and 7th documented case in the USA of *L. garviae* causing infective endocarditis of a prosthetic valve [1].

*L. garviae* is found in unpasteurized dairy products, raw fish, and meat (pork, beef, and poultry), but the route of human transmission remains unclear [3]. It seems to have a predilection for individuals with prosthetic valves, immunocompromised states, prior gastrointestinal surgery, gastrointestinal disorders (colon polyps and diverticulosis), and the use of acid-reducing medications [1-3]. Infective endocarditis is the most common systemic disease caused by *L. garviae* [1-4].

This report details the case of a 75-year-old male, with multiple comorbidities and risk factors for *L. garviae* infection who was admitted for “symptomatic anemia”. High clinical suspicion, coupled with an inadequate hemoglobin response to transfusion, a normal anemia workup, and blood cultures positive for *L. garviae*, promoted a transesophageal echocardiogram (TEE). However, the results were negative. Consequently, an ^18^F-fluorodeoxyglucose positron emission tomography/computed tomography scan (^18^FDG PET/CT) was performed. The scan revealed increased uptake in the aortic valve replacement consistent with prosthetic valve endocarditis in the setting of *Lactococcus garviae* bacteremia.

## Highlights

• Mortality caused by *L. garviae* is proportionate to streptococcus.
• Lactococcus garviae is a rare human pathogen.
• Early recognition improves overall morbidity/mortality in infective endocarditis.
• ^18^F-FDG PET/CT(A) aids diagnosis with prosthetic valves and negative TTE/TEE.
• Ultrasound and modified Duke Criterion are limited with prosthetic valves.

A 75-year-old white male with a complex medical history presented to the emergency department with general weakness, fatigue, and dizziness. His past medical history included heart failure with a preserved ejection fraction, stroke, essential hypertension, atrial fibrillation (on Warfarin), coronary artery disease, non-rheumatic aortic stenosis requiring transcatheter aortic valve replacement (TAVR) 8 months prior, insulin-dependent diabetes, gastroesophageal reflux disease, diverticulosis, chronic back pain, morbid obesity status post gastric bypass, severe peripheral arterial disease, and ruptured abdominal aortic aneurysm (previously underwent aortobifemoral-popliteal graft).

On arrival, he was hemodynamically stable and afebrile. Workup revealed significant anemia (hemoglobin [Hgb] 7.2g/dL normal range 13.2–16.6 g/dL) and a subtherapeutic prothrombin-international normalized ratio (PT/INR) of 1.6. He was admitted for further evaluation of his symptomatic anemia.

According to the initial evaluation, the patient reported fatigue, general weakness, and intermittent dizziness that had progressed over 4 weeks, affecting his daily activities. He denied bleeding but admitted to holding his Warfarin due to a supratherapeutic PT/INR of 6.2 a week prior. He described the dizziness as fleeting, non-positional, with no clear aggravating or alleviating factors. He denied other symptoms.

Due to his cardiac history and low Hgb, two units of packed red blood cells were ordered for transfusion. Notably, his baseline Hgb was typically close to 12g/dL. Additionally, admission laboratory tests included fecal occult blood testing, a complete anemia panel (including serum iron studies), inflammatory markers, thyroid levels, liver function tests, and vitamin B-12, and folate levels.

On the day following admission, vitals were as follows: blood pressure was 128/45 mmHg, heart rate 71 beats per minute, oral temperature 99.3 °F, respiratory rate 17 breaths per minute, and oxygen saturation 97 %. Weight 195lb (88.5kg) and body mass index was 31.47kg/m^2^.

The patient appeared pleasant, cooperative, pale, alert, and oriented. He was hemodynamically stable, with no signs of acute distress. A systolic ejection murmur was noted on the right sternal border. Lungs clear to auscultation. The abdomen was obese soft non-tender, and non-distended. Splinter hemorrhages were observed on both right and left index fingers. The patient had intact neurovascular function with no evidence of infection or ischemia. Examination findings did not suggest osteomyelitis, epidural abscess, discitis, cauda equina, or conus medullaris syndrome.

A review of the patient's labs revealed normocytic anemia with Hgb 7.6 g/dL despite a transfusion of two units (previous Hgb 7.2 g/dL, baseline 11.6 g/dL). Platelets decreased slightly from 210 g/dL to 186 g/dL (normal range 150–400 × 10^9^/L). Iron studies and the remainder of the anemia workup were normal except for elevated lactic dehydrogenase (LDH) at 449 U/L (normal range 135–225 g/dL for males), erythrocyte sedimentation rate (ESR) 36 mm/hr (normal range males >50 years is ≤ 20 mm/hr) and C-reactive protein (CRP) at 8.7 mg/dL (normal range <3 mg/dL) Notably, the PT/INR remained subtherapeutic at 1.6 (previously 6.2 a week before admission). Other labs were unremarkable.

The elevated LDH, ESR and CRP despite normal iron studies and no evidence of bleeding, prompted further discussion with the patient regarding the onset of other potential symptoms. These lab values are elevated in patients with sepsis [1,7].

Following this discussion, the patient revealed new atraumatic, asymmetric polyarthralgia, and an exacerbation of his chronic back pain unresponsive to his usual medication. He also admitted experiencing anorexia, unintentional weight loss of 7 lbs, nocturnal fevers, and night sweats for 7 weeks, longer than the previously mentioned 4 weeks. He initially dismissed these symptoms as irrelevant.

Considering this new information, physical examination findings, the combination of symptoms above and below the diaphragm, and the abnormal laboratory values, high clinical suspicion for infective endocarditis arose particularly given his recent TAVR 8 months prior. Blood cultures and a transthoracic echocardiogram (TTE) were ordered for further evaluation. Antibiotics were not yet initiated. While bacteremia was suspected, the patient did not meet sepsis or systemic inflammatory response criteria at that time. The team opted to monitor for potential fever.

Overnight, the patient developed a fever with a maximum temperature of 101 °F (38.3 C). Following the sepsis protocol, he underwent blood cultures from multiple sites (pan cultures) and was started in intravenous ceftriaxone 2 g every 24 hours. The following morning, preliminary results from all four blood cultures showed gram-positive cocci in pairs.

More than 72 hours after collection, final blood culture results identified *Lactococcus garviae,* an opportunistic human pathogen. Susceptibility testing revealed sensitivity to ceftriaxone but resistance to clindamycin.

Upon receiving preliminary blood culture results, the cardiology and infectious disease departments were consulted, and a transesophageal Ultrasound (TEE) was ordered to evaluate for potential infective endocarditis.

Later that afternoon during rounds, the patient again reported experiencing his previous dizziness while lying in bed. The team initially questioned whether this symptom was related to the infection. However, given the reoccurrence of this non-peripheral symptoms in the context of *L. garviae* bacteremia and recent TAVR procedure, concerns for infective endocarditis and possible septic emboli as a cause of the dizziness heightened. To investigate further, a brain computed tomography (CT) scan without contrast was performed to rule out septic emboli.

TTE and TEE revealed normal right and left ventricular function with an ejection fraction of 65 %. There was mild thickening of the mitral valve with mild regurgitation (graded +1). Mild regurgitation (graded +1) of the pulmonic valve a normal tricuspid valve, and an aortic valve replaced with an Edwards S3 prosthetic valve (#26) during TAVR. While aortic valve leaflets appeared thin with a normal opening, there was no evidence of prosthetic valve stenosis, vegetation, or abscess. Additionally, no wall motion abnormalities were observed.

A brain CT scan revealed new areas of hypoattenuation and focal sulcal effacement in the inferolateral aspect of the right frontal lobe measuring 3.5 cm × 4.2 cm. These findings are consistent with a subacute infarct. Furthermore, multiple punctate foci of hyperattenuation within the lower part of the subacute infarct are compatible with petechial hemorrhages.

Despite negative TTE and TEE findings for vegetation, the patient now met five minor Duke criteria for infective endocarditis. Additionally, the CT scan showed evidence of multiple septic petechial hemorrhages and an infarct in the setting of *L. garviae* bacteremia, raising high suspicion for infective endocarditis.

**Fig. 1:**
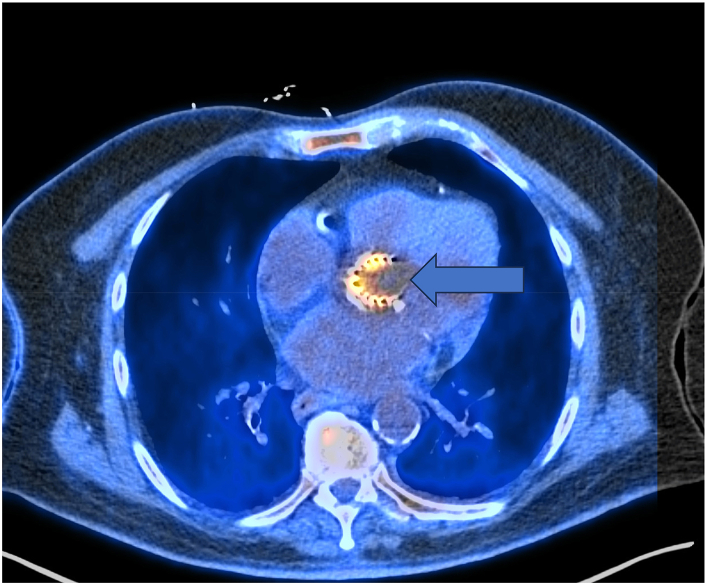
^18^F-FDG PET/CT axial fused image from the patient below depicts the uptake of the ^18^F FDG radiotracer isotope as shown by the blue arrow in the aortic valve replacement, consistent with prosthetic valve endocarditis. This intense activity shown in yellow below was present on nonattenuation corrected images and therefore, deemed not to be a valve artifact. (For interpretation of the references to colour in this figure legend, the reader is referred to the Web version of this article.)

To investigate further, an ^18^F-fluorodeoxyglucose (FDG) positron emission tomography/CT (PET/CT) scan was performed. The results revealed increased FDG activity in the aortic valve replacement activity exceeding both the reference activity in the left ventricle and the liver. This pattern as shown in Fig. 1 from the patient's PET/CT scan is consistent with a prosthetic valve inflammatory process most likely due to prosthetic valve endocarditis. Notably, this activity was intense on non-attenuated and corrected images confirming its validity and ruling out artifacts [11].

Due to the patient's high surgical risk and in the absence of valvular abscess or abnormal valve function on cardiac imaging, the team opted for conservative medical management. The recommended infectious disease regimen of intravenous IV ceftriaxone 2g every 12 hours was administered over 6 weeks. Blood cultures repeated after 24 hours of afebrile therapy and initiation of ceftriaxone were negative, indicating a good initial response to treatment. Following the completion of ceftriaxone therapy, the patient will transition to a maintenance regimen of cefdinir 300mg orally twice a day.

The patient was initially discharged to a skilled nursing facility to receive intravenous antibiotics. During post treatment follow up, he reported complete resolution of all symptoms, including his dizziness, back pain and joint pain. He regained his functional capacity and can perform his regular activities without limitations. Notably blood cultures remained negative throughout treatment, both after the initial 6-week course of ceftriaxone monotherapy and the suppressive therapy with cefdinir.

*L. garviae* is an uncommon but increasingly prevalent human pathogen. While the first case of infective endocarditis caused by *L. garviae* was reported in 1991, its presence in humans appears to be rising worldwide [2]. This opportunistic microbe can cause various systemic diseases, including osteomyelitis, peritonitis, meningitis, and infective endocarditis. Notably, *L. garviae* is a catalase negative pathogen which induces alpha hemolysis and is known well to the aquatic farming industry for causing hemorrhagic sepsis in saltwater fish [1]. This case exemplifies this risk as the patient with *L. garviae* bacteremia developed multiple septic petechial emboli identified on a brain CT scan.

Fewer than 30 documented cases of *L. garviae* induced infective endocarditis have been reported globally. Due to its similarities to Enterococcus and its prior classification within the streptococcus genus, the underdiagnosis of *L. garviae* aa a human pathogen is suspected. This opportunistic bacterium appears to have a predilection for immunocompromised individuals, particularly those with prosthetic valves, a history of gastric bypass surgery, or those taking acid-reducing medications (which may elevate gastrointestinal (GI) tract pH and facilitate bacterial growth). Additionally, *L. garviae* has been isolated from patients with various cardiovascular diseases who consume raw fish, meat, or unpasteurized dairy [[1], [2], [3], [4], [5]].

The exact source of the *L. garviae* infection remains unclear. While undercooked shrimp has been implicated in some cases, the patient frequently consumed it before the symptom onset and even several months after his TAVR procedure. Further investigation revealed he had undergone a dental cleaning around the same times as his symptoms, receiving clindamycin prophylaxis. It is possible that the combination of these factors along with his history of gastroesophageal reflux disease, bariatric surgery, diverticulosis, and multiple cardiovascular conditions with prior surgeries (particularly his aortic valve replacement), created a suitable environment for the pathogen to establish itself.

Another potential contributing factor is the patient's chronic use of acid-reducing medications. Studies suggest these medications may weaken the natural barrier in the gut, promoting bacterial growth [16]. Given his significant GI medical history, outpatient follow-up with a gastroenterologist for endoscopy and colonoscopy was recommended, along with other evaluations deemed necessary. While an association between *L. garviae* and various GI disorders exists, the rarity of this organism causing severe human disease makes it unclear if a link to GI carcinoma exists [16].

Unlike most reported cases of *L. garviae* prosthetic valve endocarditis, this patient did not present with acute or subacute fever or altered mental state. While initially evaluated for symptomatic anemia, his presentation shifted to a concern for infective endocarditis due to multiple vague constitutional symptoms above and below the diaphragm reported over 7 weeks and months following his TAVR procedure. Additionally, his physical examination did not correlate well with these symptoms. Notably, his hemoglobin level remained unchanged despite transfusions. The cause of the anemia remained unclear, and his renal function was normal. He developed a fever his second night in hospital.

Based on these concerns, blood cultures, TTE, additional labs ordered, and cardiology and infectious disease consultations were ordered and performed. Both TTE and subsequent TEE yielded negative results. However, final blood cultures ultimately identified *L. garviae*.

Furthermore, the patient's dizziness in the absence of peripheral symptoms, mildly elevated LDH and inflammatory markers coupled with positive cultures, maintained infective endocarditis as a top diagnosis. A brain CT brain was performed to evaluate for possible septic emboli and infarct, fulfilling the five minor Duke criteria for infective endocarditis: splinter hemorrhages, septic emboli with infarct, fever, a predisposing heart condition and positive blood cultures (although *L. garviae* is not classified as major HAECK criteria microbe).

Given these findings, the treatment team ordered an ^18^F-FDG PET/CT scan. This scan revealed increased radiotracer uptake in the aortic valve replacement, consistent with prosthetic valve endocarditis.

Traditionally, TTE followed by TEE is the standard diagnostic imaging approach for infective endocarditis. However, for patients with prosthetic valves and other intracardiac devices the effectiveness of ultrasound and modified Duke criteria may be limited [7]. A prospective study by Pizzi et al. suggests that PET CT and PET CT angiography (PET/CTA) offer greater sensitivity and specificity in this patient population [2,6,7]. This case supports these findings and highlights the potential limitations of echocardiography in patients with cardiac devices where device artifacts can obscure vegetations. As suggested by previous authors utilizing PET/CT and PET/CTA can offer greater sensitivity as demonstrated in this case [2,6,7].

There is currently no standardized diagnostic, or treatment guidelines for *L. garviae* infective endocarditis. However, due to its similarities with streptococcal infections, monotherapy options include ceftriaxone, vancomycin, amoxicillin, and ampicillin. Dual therapy with gentamycin is also used. Notably, this patient's *L. garviae* strain exhibited resistance to penicillin and clindamycin [[1], [2], [3],[7], [8], [9], [10]].

Ceftriaxone was chosen for this patient due to its favorable side effect profile, ease of administration, and good tolerability. The patient responded well to treatment and transitioned to lifelong maintenance therapy with cefdinir 300mg orally twice daily. Close monitoring by multidisciplinary team is ongoing. Suppressive maintenance therapy was selected for this patient due to high risk of recurrence. This risk stems from his aforementioned risk factors and his inelligibility for surgical intervention.

Prosthetic valve replacements are a risk factor for infective endocarditis, accounting for approximately 20% of diagnosed cases and affecting 6% of patients who undergo the procedure [7,8]. *L. garviae* endocarditis typically presents sub-acutely and mortality is comparable to streptococcal infective endocarditis (approximately 16–20 %) [9].

Therefore, early recognition of the constitutional symptoms in high-risk patients is crucial. When clinical suspicion is high and ultrasound is inconclusive, ^18^F-FDG PET/CT can be a valuable tool to aid in the diagnosis of infective endocarditis. Early identification, diagnosis, and treatment can significantly improve patient outcomes [[6], [7], [8], [9], [10], [11], [14], [15], [17], [18]].

## Ethical statement

We hereby confirm that we have read and complied with the policy on ethical conduct.

All participants/patients (or their proxies/legal guardians) provided informed consent for the publication of their anonymized case details and images.

## Funding

No funding was provided for the above case report.

## Data availability

Has data associated with your study been deposited into a publicly available repository?

No. All data pertinent to the case is presented in this publication with verbal and written patient consent.

## CRediT authorship contribution statement

**Nicole Schtupak:** Conceptualization, Data curation, Formal analysis, Funding acquisition, Investigation, Methodology, Project administration, Resources, Validation, Visualization, Writing – original draft, Writing – review & editing. **Patrick Kenney:** Investigation, Resources. **Darko Pucar:** Resources. **Linda Godinez:** Investigation, Resources. **Jodi-Ann Chin:** Investigation, Resources. **Kristen Selema:** Resources. **Dipan Uppal:** Resources. **Antonio Lewis:** Resources. **Marcelo Helguera:** Resources.

## Declaration of competing interest

The authors declare that they have no known competing financial interests or personal relationships that could have appeared to influence the work reported in this paper.
